# Creatine deficiency and heart failure

**DOI:** 10.1007/s10741-021-10173-y

**Published:** 2021-10-07

**Authors:** Annamaria Del Franco, Giuseppe Ambrosio, Laura Baroncelli, Tommaso Pizzorusso, Andrea Barison, Iacopo Olivotto, Fabio A. Recchia, Carlo M. Lombardi, Marco Metra, Yu F. Ferrari Chen, Claudio Passino, Michele Emdin, Giuseppe Vergaro

**Affiliations:** 1 Institute of Life Sciences, Scuola Superiore Sant’Anna, Pisa, Italy; 2 Division of Cardiology, School of Medicine, University of Perugia, Perugia, Italy; 3 Institute of Neuroscience, National Research Council (CNR), Pisa, Italy; 4 Department of Developmental Neuroscience, IRCCS Stella Maris Foundation, Calambrone, PI Italy; 5 Department of Neuroscience, Drug Research, and Child Health (NEUROFARBA), University of Florence, PsychologyFlorence, Italy; 6 Division of Cardiology and Cardiovascular Medicine, Fondazione Toscana Gabriele Monasterio, Pisa, Italy; 7 Cardiomyopathy Unit, Azienda Ospedaliera Universitaria Careggi, Florence, Italy; 8 Lewis Katz School of Medicine, Cardiovascular Research Center, Temple University, Philadelphia, PA USA; 9 Department of Medical and Surgical Specialties, Radiological Sciences and Public Health, University and Civil Hospital, Brescia, Italy

**Keywords:** Creatine, Creatine deficiency, Cardiac energy metabolism, Heart failure

## Abstract

Impaired cardiac energy metabolism has been proposed as a mechanism common to different heart failure aetiologies. The energy-depletion hypothesis was pursued by several researchers, and is still a topic of considerable interest. Unlike most organs, in the heart, the creatine kinase system represents a major component of the metabolic machinery, as it functions as an energy shuttle between mitochondria and cytosol. In heart failure, the decrease in creatine level anticipates the reduction in adenosine triphosphate, and the degree of myocardial phosphocreatine/adenosine triphosphate ratio reduction correlates with disease severity, contractile dysfunction, and myocardial structural remodelling. However, it remains to be elucidated whether an impairment of phosphocreatine buffer activity contributes to the pathophysiology of heart failure and whether correcting this energy deficit might prove beneficial. The effects of creatine deficiency and the potential utility of creatine supplementation have been investigated in experimental and clinical models, showing controversial findings. The goal of this article is to provide a comprehensive overview on the role of creatine in cardiac energy metabolism, the assessment and clinical value of creatine deficiency in heart failure, and the possible options for the specific metabolic therapy.

**Supplementary information:**

The online version contains supplementary material available at 10.1007/s10741-021-10173-y.

## Biochemistry of creatine

**Fig. 1:**
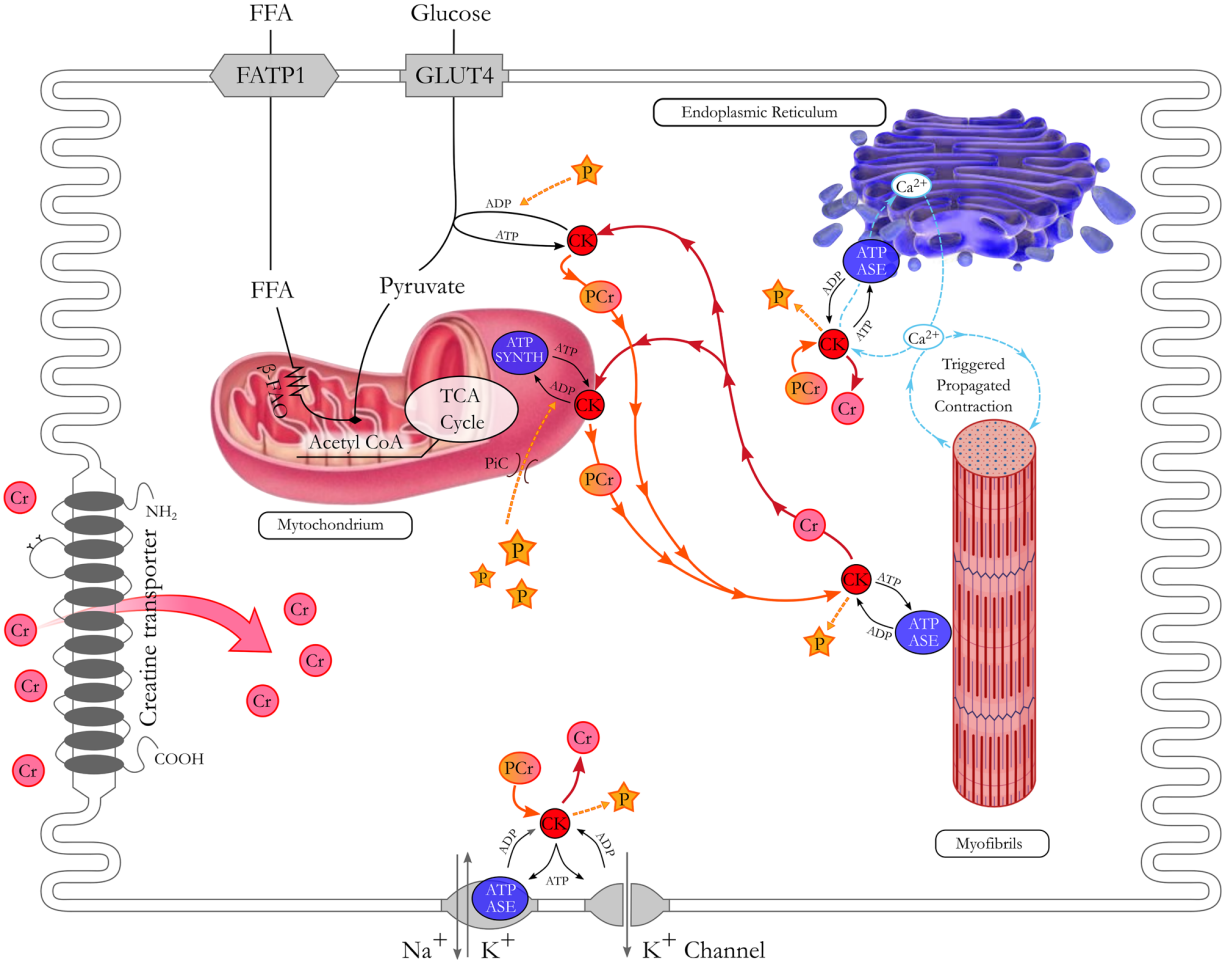
Role of creatine in the cardiomyocyte. A specific carrier (CrT) mediates creatine (Cr) uptake from bloodstream into cardiomyocytes. Cr links adenosine triphosphate (ATP) production site to energy utilization sites, like myofibrils and ion pumps. Phosphocreatine (PCr) acts as a highly mobile and short-term energy store. After releasing the phosphate group to generate ATP thanks to the cytosolic creatine kinases (CK) closely coupled to ATPases, free Cr diffuses back to request further ATP production. β-FAO beta fatty acid oxidation, FATP1 fatty acid transport protein 1, FFA free fatty acid, GLUT4 glucose transporter type 4, PiC mitochondrial phosphate carrier, TCA tricarboxylic acid

Creatine (Cr) is a guanidino compound that plays a vital role in the energy metabolism of cells. Cr kinase (CK) catalyses the reversible conversion of Cr and adenosine triphosphate (ATP) to phosphocreatine (PCr) and adenosine diphosphate. Many cells do not rely on ATP/adenosine diphosphate free diffusion and the CK/PCr/Cr system servers as energy storage for immediate regeneration of ATP and as a shuttle of high-energy phosphate between the mitochondrial site of ATP production and cytosolic sites of ATP consumption [1] (Fig. 1). Specifically, the CK/PCr system serves 3 major functions: “temporal” energy buffer, employing PCr and Cr to maintain adequate ATP concentrations; “spatial” energy buffer, creating tightly coupled connections between ATP-producing and ATP-consuming processes; and regulation of metabolic pathways. Under physiological conditions, the CK system is predominant and provides about two-thirds of the energy transfer, whereas the direct adenine nucleotide channelling contributes only to one-third [2]. Accordingly, Cr is particularly abundant in tissues with high and intermittent energy fluctuations, such as skeletal muscle, heart, and brain [1].

**Fig. 2:**
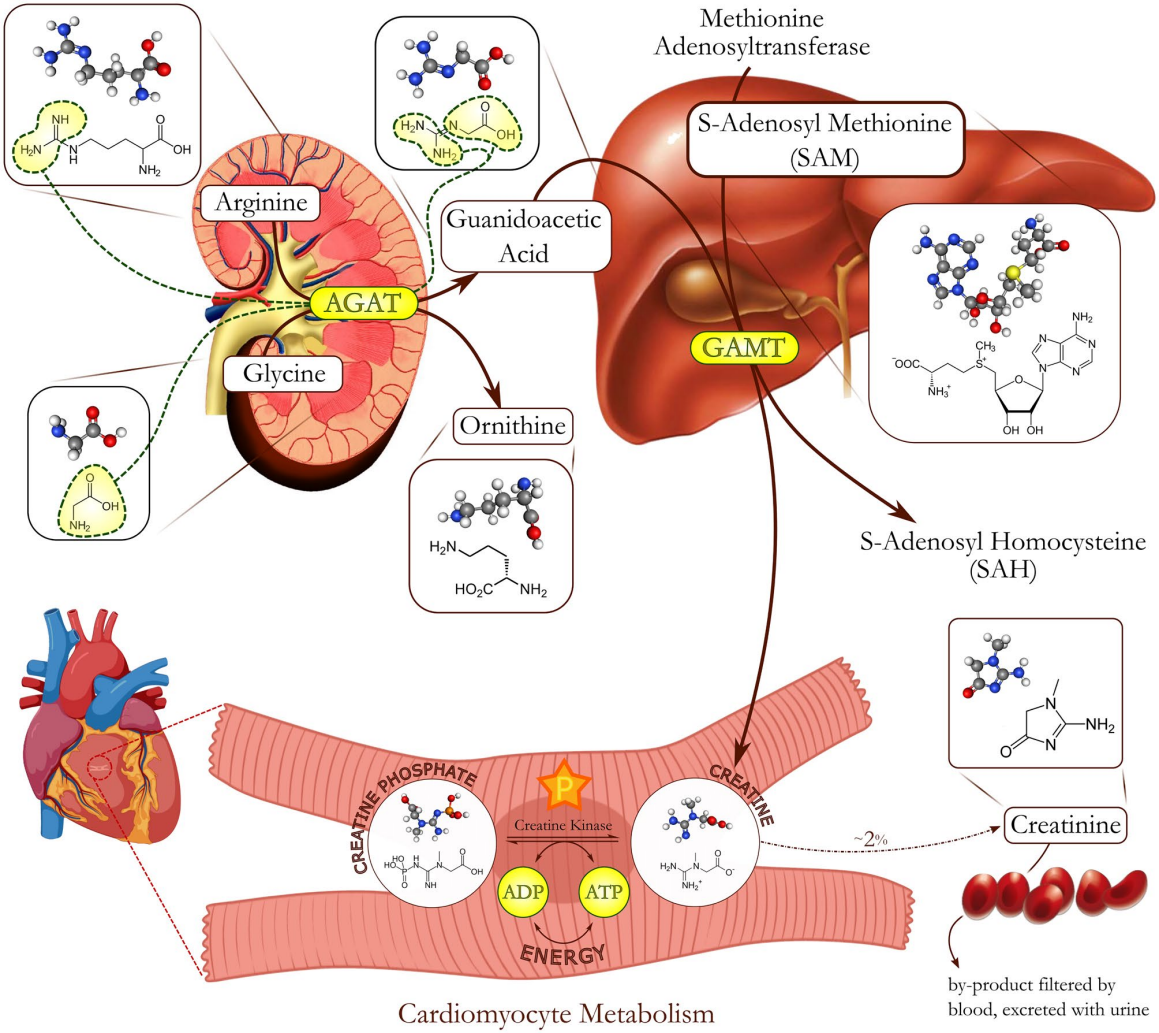
Creatine biosynthesis. Creatine 2-step biosynthesis: the transfer of an amidino group from arginine to glycine, catalysed by L-arginine:glycine amidinotransferase (AGAT), yields the guanidinoacetate (GA); GA is converted into Cr via the enzyme S-adenosyl-L-methionine:guanidinoacetate N-methyltransferase (GAMT). The first step occurs in the kidney, the second in the liver. Cr and phosphocreatine (PCr), together with creatine kinase (CK), constitute an energy shuttle system. Cr degrades into creatinine, excreted by the kidney

Cr is endogenously synthesized and absorbed from dietary sources, then mainly stored in muscles. Endogenous biosynthesis involves 2 sequential steps: first, the transfer of an amidino group from arginine to glycine, catalysed by L-arginine:glycine amidinotransferase (AGAT), yields the intermediate guanidinoacetate (GA); GA is then converted into Cr via the enzyme S-adenosyl-L-methionine:guanidinoacetate N-methyltransferase (GAMT) [3] (Fig. 2). GA is primarily produced in the kidney and methylated to Cr in the liver; then, Cr is released in the bloodstream to be delivered throughout the body [3]. Cr is a polar hydrophilic molecule unable to cross the plasma membrane; hence, it requires a specific Na^+^/Cl^−^-dependent transporter (Cr transporter, CrT; solute carrier, SLC6A8) to enter the cells [4]. Cr transport is largely regulated by substrate availability: the increase in extracellular Cr content reduces the rate of Cr uptake, whereas decreased intracellular Cr content increases Cr transport. Although there is a unique CrT protein, its modulation is complex and tissue-specific. With this regard, thioredoxin interacting protein has been identified as an important negative-feedback regulator, acting as a promoter of intracellular Cr concentration [5]. Furthermore, supporting the idea that Cr transport might be modulated by pathways that respond to changes in the overall cellular metabolic state, 5-AMP-activated protein kinase has shown to be a positive and physiological regulator of Cr transport, ensuring sufficient quantities of Cr and supporting myocellular function [6]. Similarly, several analyses in murine models have shown that in cardiac muscle, modulation of Cr transport capacity is primarily regulated by post-translational modifications of CrT protein, rather than by transcriptional changes [7].

Finally, Cr and PCr are subject to a slow, non-enzymatic, and unregulated dehydration and cyclisation yielding creatinine (about 1.7% of Cr/PCr body pool): this end-product freely diffuses out the cell to be ultimately removed with urine.

## Physiology of creatine and phosphocreatine in cardiomyocytes

Chemical energy in the form of ATP is produced from multiple substrates (principally long-chain fatty acids, lactate, and glucose), predominantly in the mitochondria via oxidative phosphorylation. To ensure that energy is readily available at sites of utilization, a phosphagen system is required, consisting in the reversible interaction of Cr and ATP under the control of CK enzymes [8]. Tissue- and cell-specific CK isoforms are well known. At cardiac level, muscle-type CK and mitochondrial CK are the most represented [9]. Mitochondrial CK catalyses direct transfer of a high-energy phosphoryl group from ATP to Cr to form PCr, acting as a highly mobile, short-term energy store, as it is smaller and less polar than ATP [3]. The reverse reaction is catalysed by the cytosolic CK dimers and generates ATP; then, the free Cr diffuses back to signal the need for further ATP production [9].

In addition to its role in ATP regeneration, PCr acts as membrane stabilizer [10]. PCr may therefore promote the transition of the mobile domain of membranes into a structured phase, leading to a decreased rate of phospholipids degradation into lysophospholipids and lipid peroxidation. This second mechanism is particularly relevant for cardiac function, since the PCr/Cr plays a protective role against ischaemic insults [10].

## Assessment of cardiac creatine metabolism

Magnetic resonance spectroscopy (MRS) is the only technique capable to non-invasively measure the ratios and concentrations of endogenous cardiac high-energy phosphate metabolites and CK flux in human hearts. Cardiac MRS is a challenging technique, since the heart is constantly in motion (ECG-gating, breath-holding) and due to the very low concentration of nuclei of interest (^31^P, ^13^C) if compared to ^1^H.

MRS has been first technology employed to obtain the measure of myocardial PCr/ATP ratio [11]. MRS can be used to measure the absolute concentrations of PCr and ATP, the CK reaction rate, and flux [12] by assessing phosphorus (^31^P MRS), as well as to measure total Cr pool by assessing proton (^1^H MRS) [13]. Combining 31P and 1H MRS, it is possible to characterize CK metabolism in the healthy and diseased human heart [14].

The PCr/ATP ratio has been used as a marker of cardiac energetic status in heart failure (HF). Specifically, PCr level decreases in the initial phase, whereas ATP level decreases only in advanced stages of HF. Myocardial PCr, Cr, and CK enzyme levels are consistently low in HF, and can precede cardiac dysfunction [15], while they are preserved in other heart diseases such as ischaemic heart disease [11]. The PCr/ATP ratio, however, is not affected when proportional changes in both PCr and ATP concentrations occur. Beer and colleagues [16] observed that PCr and ATP concentrations are both reduced in dilated cardiomyopathy, although with a greater reduction in PCr than in ATP. Thus, the PCr/ATP ratio alone may underestimate the extent of changes in high-energy phosphate levels, especially during the earlier stages of HF. Furthermore, experimental studies have earlier documented that the assessment of PCr and ATP cardiac concentrations might provide little information at the steady-state. In fact, if the energy machinery remains functional, as in the “stunned” heart, PCr and ATP can be re-synthesized at a suitable rate to support an increased demand [17].

**Fig. 3:**
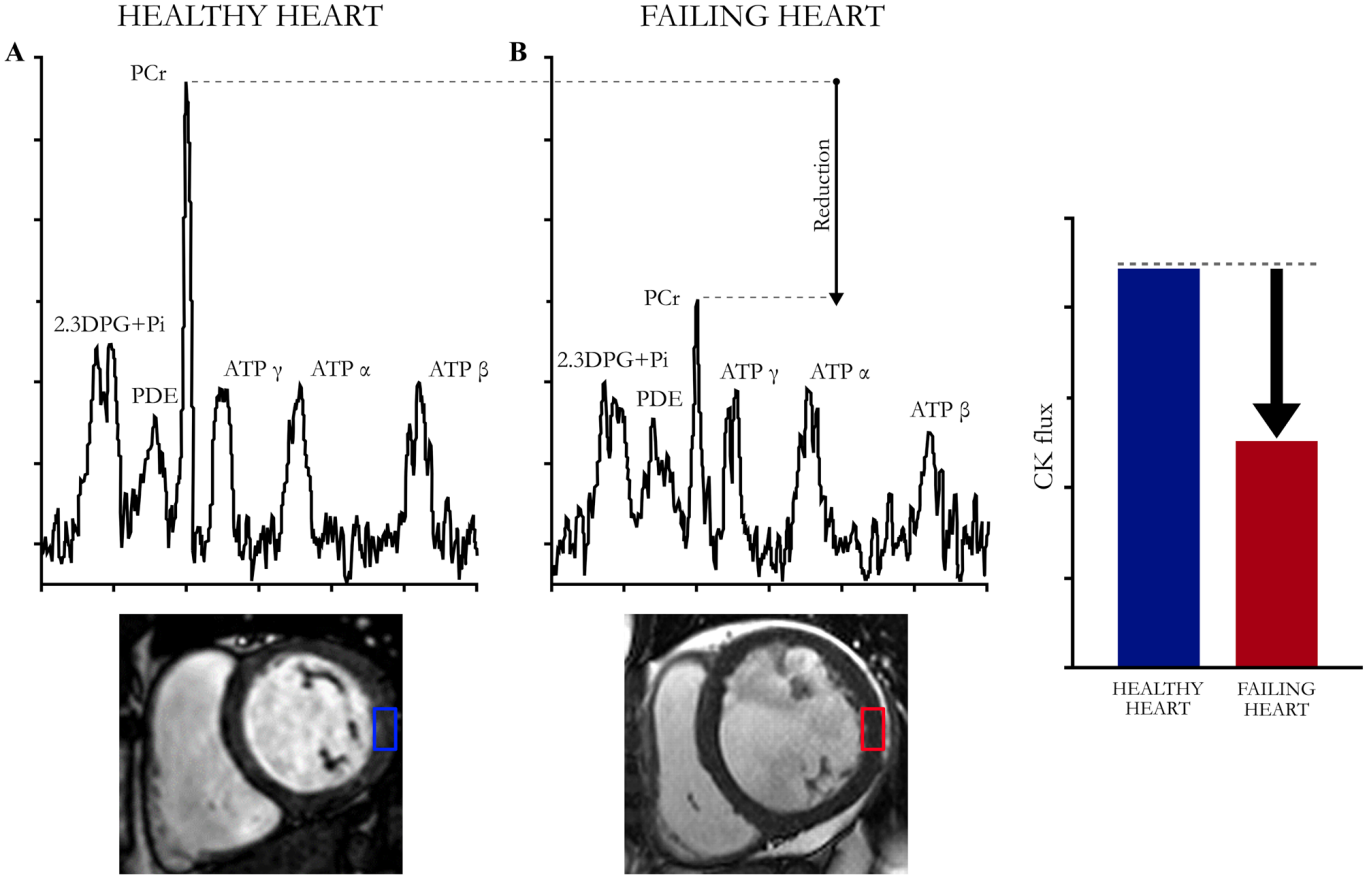
31P magnetic resonance spectroscopy (MRS) cardiac evaluation. Characteristic cardiac 31P MRS spectra in **A** a healthy and **B** failing heart. In the pathological condition, PCr concentration and CK flux are reduced

Since the PCr/ATP ratio cannot provide reliable information about the effective availability of high energy phosphates, other MRS indexes were developed. The product of the pseudo-first-order forward reaction-rate constant for the CK reaction (kf) and the PCr concentration (CK flux) allows the measurement of the forward rate of ATP synthesis through CK system [18] (Fig. 3). Studies combining assessment of PCr and ATP concentration and CK flux demonstrated in non-ischaemic dilated cardiomyopathy not only that PCr concentration is reduced, but also that the reduction in CK flux (∼35 to 70%) is greater than the reduction in the PCr/ATP ratio (∼10 to 20%) [12]. Thus, the reduced ATP supplied by the CK reaction, rather than the increase in adenosine diphosphate (ADP) concentration, might contribute to the dysfunction. Another study showed that only CK flux is capable of predicting outcomes in non-ischaemic dilated cardiomyopathy [19]. Additionally, the loss in CK energy reserve matches the reduction in mechanical efficiency, pointing to inadequate energy supply as a potential contributor to the contractile dysfunction observed in mild-to-moderate HF. Finally, since CK flux reduction is more pronounced than PCr/ATP ratio reduction [12], it seems more reliable and accurate in predicting outcomes and mortality even in earlier stages of HF [20].

The severity of the energetic deficit was also evaluated by determining the free energy of ATP hydrolysis, which is the driving force that powers cellular ATP-requiring processes. The quantification in vivo of cardiac Pi concentration by higher-field MRS systems allows the calculation of ATP hydrolysis. In failing heart, the decrease in Cr concentration is initially thought to be a compensatory mechanism counteracting the expected increase in cytosolic ADP, with consequent preservation of the free energy of ATP hydrolysis [21]. However, in the scenario of high ATP consumption, such as during exercise or stress, once CK activity is decreased, the higher ATP and ADP fluctuations reduce the energy available from ATP hydrolysis, limiting the maximum available energy during peak demand [22].

MRS use is currently limited to research purposes, but it might improve our understanding of the pathophysiology of several cardiac conditions. Technical advances are still required for the clinical use of cardiac MRS.

## Creatine deficiency in heart failure

According to the energy starvation hypothesis, altered myocardial metabolism anticipates and sustains contractile dysfunction [23]. The healthy heart has a high capacity for ATP turnover and, during exercise, cardiac output and O_2_ consumption may rise up to sixfold. This functional reserve is granted by a considerable number of mitochondria, balancing the limited amount of stored ATP and of phosphotransfer systems. In HF, contractile reserve is limited both by an impairment of PCr/CK system [24] and by mitochondrial dysfunction [25].

Decreased cardiac Cr content and total CK activity are observed in HF regardless of underlying aetiology, prominently in advanced stages [26]. Additionally, low PCr is an early indicator of an energetic deficit [24] and PCr/ATP ratio is associated with more severe HF symptoms [11], low contractile function, and myocardial structural remodelling [27], proving to be an excellent predictor of cardiovascular mortality in patients with dilated cardiomyopathy [28]. Cr loss is likely to occur secondary to a reduced expression of the CrT [29]. However, it is not completely clear to what extent the reduced PCr buffer activity contributes to the pathophysiology of HF and if a correction of this energetic deficit would prove beneficial.

The functional role of high-energy phosphate metabolism in HF should be investigated focusing on the depletion of myocardial PCr and Cr levels. A way to achieve that condition could be by means of a pharmacological compound known to specifically reduce PCr and Cr in the heart, the β-guanidinopropionate. Specifically, β-guanidinopropionate is a Cr analogue that is taken up by the cardiomyocyte via the CrT and is then phosphorylated, causing the inhibition of CK reaction [30]. In normal heart, the depletion of PCr and Cr due to β-guanidinopropionate treatment impaired the capability of CK system to ensure high levels of sustained contractile function [31]. In ischaemic condition, the β-guanidinopropionate-induced depletion of PCr (65%) and reduction of CK flux (75%) make the heart unable to overcome the stress of an acute myocardial infarction; specifically, this finding might be due either to the remaining intact myocardium not being able to face the acute haemodynamic stress or to the induction of fatal ventricular arrhythmias — or both. However, chronic PCr and Cr depletion, obtained by β-guanidinopropionate treatment started after the recovery from coronary ligation, did not further impair myocardial performance, suggesting an adaptation of the chronically failing heart and highlighting the functional relevance of the PCr-CK system only in acutely induced HF [32]. However, β-guanidinopropionate has many limitations such as off-target effects, slow and incomplete creatine depletion, and the ability to partially compete in the creatine kinase reaction [33].

Genetically manipulated mouse models circumvent limitations of β-guanidinopropionate and provide a more accurate approach to assess the functional significance of the PCr/CK system. In particular, genetic loss-of-function mice have been described for all CK isoforms, for Cr-related enzymes (AGAT, GAMT) and for CrT. Even if muscle/mitochondrial CK^−/−^ mice show elevated end-diastolic pressure, impaired contractility, and pulmonary congestion [34], a primary deficiency of CK system does not determine per se an overt HF [35], but rather a cardiac phenotype that can be unmasked during acute stress conditions. Moreover, a genetically determined reduction of CK function is likely to result in long-term compensatory adaptations, which cannot be set during acute cardiac damage [34]. Cardiac functions of AGAT and GAMT have been studied in global knockout mice, since these enzymes are not expressed in the heart. In the AGAT^−/−^ model, only low LV systolic pressure seems to be related to Cr depletion, whereas other haemodynamic alterations such as the reduced inotropy and the impaired contractile reserve are rescued after homoarginine supplementation [36]. Similarly, GAMT^−/−^ model shows only a small reduction in LV systolic pressure and an impaired contractile reserve at baseline [37]; these alterations do not have consequences in terms of exercise performance and of cardiac function after myocardial infarction in Cr-deficient condition [38]. In particular, if the unaltered cardiac function at “resting” condition suggests that the CK/PCr system does not play a crucial role in maintaining cardiac performance under low workload, on the opposite, the absence of a high-capacity CK/PCr system seems to aggravate myocardial injury in case of acute myocardial stress due to energy demand/supply mismatch [37]. These findings suggest a possible redundancy of energy buffer and transfer systems at baseline and low workload. In fact, GAMT^−/−^ mice receiving a Cr-free diet accumulate GA, which can potentially compensate the Cr deficiency by participating in the CK reaction [38].

Mechanisms underlying the compensatory condition and their long-lasting effects are under investigation. There is evidence that 18 months old GAMT^−/−^ mice have reduced rates of contractility and relaxation [39]. This finding is not clearly related to the Cr deficiency or the to the GA toxicity, but might suggest that adaptations observed in young mice are subject to age-related decline [39].

The phenotypic characteristics at cardiac level of a CrT^−/y^ murine model have not been described yet, despite a reduced cardiac Cr concentration has been shown [40]. While Cr supplementation is not effective to increase intracellular Cr content due to the downregulation of CrT, the augmentation of the CrT activity is expected to be beneficial [8]. Increasing myocardial Cr levels by 20–100%, via overexpression of the plasma membrane CrT, protects the murine heart against ischaemia/reperfusion injury through the improvement in energy reserves and increased PCr and glycogen levels [41]. However, no benefit in terms of cardiac remodelling and function was detected in a model of post-myocardial infarction chronic HF. Moreover, in case of very high cardiac Cr concentrations exceeding 100% of total increase, severe cardiac hypertrophy and dysfunction are reported [42].

In conclusion, the PCr-CK system seems essential for supporting the function of hearts in healthy and in high energy demand or acute stress, whereas it loses its importance in chronic condition, probably due to the emergence of compensatory mechanisms. The interruption of a single step of the metabolic chain is probably not sufficient to mimic the complex interactions occurring in chronic HF.

## Exercise training and creatine kinase system

Training increases exercise capacity, quality of life, and reduces morbidity and mortality in HF by improving endothelial function and coronary perfusion, decreasing peripheral resistance and ventricular remodelling, as well as neuro-endocrine and pro-inflammatory activation [43].

The positive effects of exercise on cardiac energy metabolism, in particular on the Cr system, are not well-understood. An improvement in oxidative capacity and a restoration of CK activity after exercise training has been reported [44], confirming that the CK system actively takes part to the intracellular metabolism. On the opposite, GAMT knockout mice display exercise performance and survival similar to wild type mice [38], questioning if Cr is essential for high workload and chronic stress responses in heart and skeletal muscle.

Although preliminary evidence exists supporting an improvement in pro-inflammatory drive and endothelial dysfunction by the combination of Cr supplementation and regular exercise [45], further investigations are needed.

## Pharmacological approaches to improve cardiac metabolism in heart failure

Neurohormonal antagonists are the cornerstone of pharmacological therapy of HF. Since neuro-hormonal activation significantly contributes to the metabolic inefficiency of the heart [46], an indirect beneficial effect of neurohormonal antagonists on energy efficiency might be expected. However, although both beta-blockers and angiotensin-converting enzyme inhibitors increase PCr content, CK reaction velocity and mitochondrial CK activities also reduce free fatty acid availability, a state of low metabolic efficiency in both skeletal and cardiac muscles still persists in HF [47].

Pharmacological modulation of cardiac metabolism in HF was proposed in the early 1990s [48], based on 3 mechanisms: reduction of ATP availability, decreased ATP transfer and availability to the myofibrils, and reduction of the free energy change of ATP hydrolysis.

Options to modify substrate utilization, shifting the myocardial substrate from free fatty acid metabolism to glucose metabolism, have been largely investigated [49]. Myocardial glucose oxidation can be achieved either directly with stimulation of glucose metabolism, or indirectly through inhibition of fatty acid beta-oxidation. Trimetazidine inhibits fatty acid oxidation by blocking 3-ketoacyl-coenzyme A thiolase and preserves myocardial high-energy phosphate intracellular levels [50, 51]. Similarly, ranolazine enhances myocardial utilization of glucose and significantly improves LV performance in experimental models of HF [52].

Cr supplementation showed to improve skeletal muscle mass and strength in chronic HF, while cardiac contractility was not affected [53], despite Cr induces better adaptive physiological responses mediated by hypoxia-inducible factor-1 and by the subsequent rise of cellular ATP and PCr content [54, 55]. Fumagalli et al. demonstrated an improvement in total work capacity and peak oxygen consumption validating the positive effect of supplementation of Cr in combination with coenzyme Q10, which however could be related to the beneficial effect on the diseased skeletal muscle rather than to a direct action on the myocardium [56]. With this regard, in the history of treatment strategies for HF — a status characterized by muscle wasting and cachexia — the improvement of clinical status by enhancing muscular performance has also been noticed in case of other micronutrients, hence, the idea of a multiple micronutrient approach or a combination of nutrient supplementation and exercise training [57]. In fact, Hemati and colleagues showed that the combination of Cr monohydrate and exercise attenuates inflammation and endothelial dysfunction markers in HF [45].

The unknown mechanisms regulating the organ distribution of the total Cr pool could explain the lack of effect of Cr supplementation on surrogate markers of cardiac performance. The energy-demanding heart has a lower Cr intake compared to the liver. Further, tissues with high baseline Cr (i.e. heart) have less loading potential than tissues with low Cr (i.e. liver), possibly as a consequence of CrT inhibition.

The questionable results concerning the benefits of Cr therapy in HF might also be related to the use of the sole Cr monohydrate, whether other Cr forms are known to be more efficient in terms of bioavailability and tolerability [58]. Furthermore, an indirect benefit could derive from supplementation of other components of the Cr synthesis production chain, such as L-arginine that proved to be efficacious in augmenting peripheral blood flow and improving functional status in a small cohort of HF patients [59].

Exogenous PCr has gained considerable attention as an effective and safe protective agent in different clinical settings, including cardiac surgery, myocardial infarction, and HF [60]. Treatment with PCr in patients with acute and chronic heart diseases might reduce all-cause short-term mortality and is associated with an improvement in left ventricular ejection fraction in chronic HF and with a decrease in arrhythmic complications and left ventricular remodelling in patients undergoing cardiac surgery [61] (Central illustration). However, PCr studies are based on a small population and with the absence of long-term follow up [60]. The cardiac effect of PCr supplementation might be dependent on the route of administration, as intravenous route appears to be the only yielding significant and long-lasting Cr elevation in myocardium. Moreover, differently from Cr, PCr is not carried within the cell by CrT; it does not easily cross membranes and its intracellular levels do not modulate CrT activity [62].

An overview of the principal clinical studies on Cr and PCr supplementation is illustrated in Table 1.

**Table 1:** Clinical studies on supplementation of creatine (Cr) or phosphocreatine (PCr) in patients with acute or chronic heart failure (HF). NYHA, New York Heart Association; RCT, randomized clinical trial. References provided in Supplemental Material

Author and year	Type of study	Clinical setting	Cr/PCr dose	Results
**Creatine supplementation**				
Andrews R. (1998)	Controlled trial	Chronic HF: 20 pts	20 g os daily for 5 days	- Increase in skeletal muscle endurance
Carvalho A. P. (2012)	RCT	HF (NYHA II-IV): 33 pts	5 g os daily for 6 months	- No change in functional capacity at cardiopulmonary exercise test and 6-min walking test
Cornelissen V. A. (2010)	RCT	Coronary artery disease or chronic HF: 70 pts	5 g os daily for 3 months, in combination with exercise training	- No improvement in physical performance, quality of life than exercise training alone
Fumagalli S. (2011)	RCT	Chronic HF: 67 pts	Coenzyme Q10 (320 mg) + Cr (340 mg) os daily for 8 weeks	- Improvement in total work capacity and peak oxygen consumption
Gordon A. (1995)	Controlled trial	Chronic HF: 17 pts	20 g os daily for 10 days	- No improvement of ejection fraction - Increase in muscle strength and endurance
Kuethe F. (2006)	Controlled trial-crossover trial	Acute HF: 20 pts	20 g os daily	- Increase in muscle strength - No change in peak VO2, walking distance, quality life assessment, and ejection fraction
**Phosphocreatine supplementation**				
Andreev N. A. (1992)	RCT	Acute HF: 67 pts	8 g iv in 200 ml saline at a rate of 4 g/h once a day for 21 days plus diuretic or 8 g iv in 200 ml saline at a rate of 4 g/h once a day for 21 days plus diuretic and digoxin	- Increase in left ventricular ejection fraction - Decrease in systemic vascular resistance - Decrease in ventricular arrhythmias - Decrease in the severity of dyspnoea, frequency of angina attacks - Improvement in exercise tolerance
Cafiero M. (1994)	Open clinical trial	HF (NYHA class II-III): 23 pts	5 g iv bolus administration (acute) or 5 g iv for 6 days	- Amelioration of all indexes of cardiac contractility (wall stress, ejection fraction, and fractional shortening)
Du X. H. (2009)	RCT	Elderly patients with chronic HF: 40 pts	3 g iv in 200 ml glucose 5% once a day for 8 weeks	- Reduced end-systolic and end-diastolic diameters, ejection fraction and B-type natriuretic peptide levels
Ferraro S. (1996)	Controlled trial-crossover trial	Acute HF from ischaemic heart disease or dilated cardiomyopathy (NYHA class II-III): 13 pts	6 g iv diluted in 50 ml of NaCl 0.9% acutely or 6 g infusion for 4 days (short-term treatment)	- Increase in ejection fraction and fractional shortening - Reduced end-systolic diameter and systemic vascular resistance
Grazioli I. (1989)	RCT	Chronic HF: 1174 pts	1 g iv slow infusion every 12 h during 7–21 days	- Improvement of clinical symptoms (dyspnoea, pulmonary stasis, and peripheral oedema), and signs of ischaemia (angina, use of sublingual nitroglycerin, negative T-waves)
Grazioli I. (1992)	RCT	HF: 1007 pts	1 g iv infusion every 12 h for 2 weeks, followed by 500 mg intramuscular daily administration for 1 month	- Improvement of clinical symptoms (dyspnoea, pulmonary stasis, and peripheral oedema), the main signs of ischaemia (angina, number of patients taking sublingual nitroglycerin, negative T wave), and the incidence of ventricular arrhythmias
Wang F. R.(2006)	RCT	Chronic HF (NYHA class III-IV): 64 pts	2 g iv in 100 ml saline for 14 days	- Improvement in left ventricular ejection fraction, stroke volume, and cardiac output - Lowering in B-type natriuretic peptide levels
Ying W. (2013)	RCT	Hypertensive cardiac diastolic dysfunction	2.0 g iv plus losartan-hydrochlorothiazide	- Lower systolic and diastolic blood pressure - Improvement of the diastolic dysfunction

Chemically modified, highly lipophilic Cr (cyclocreatine or Cr esters) may exploit passive diffusion across the plasmatic membrane and overcome the CrT down-regulation related to an elevated intracellular Cr content [8]. Furthermore, cyclocreatine is much more stable and is a superior long-acting phosphagen compared to PCr, since it longer sustains ATP synthesis during ischaemia by continuously phosphorylating ADP [63]. Although cyclocreatine has yielded positive effect on brain deficits in CrT knock-out mice [64], in a cardiac ischaemia/reperfusion model [65] and in cardiac surgery with a cardioprotective effect [66], its use is limited by pharmacological off-target effects and by a lower affinity for CK compared to Cr. Moreover, since the use of cyclocreatine is limited by its water insolubility and the need to be administered much earlier than the ischemic event, a new soluble preparation has been proposed and needs further investigation [63].

## Conclusions

As a component of the energy buffer system, Cr is involved in cardiac metabolism to transfer energy from site of production to site of utilization. While a decreased cardiac Cr content is a feature of advanced HF, reduction in the rate of ATP synthesis through CK system might explain contractile dysfunction and poor outcomes in HF patients even in earlier stages. Nevertheless, the effects of Cr analogue and PCr supplementation are controversial and probably need to be tested in randomized studies on top of current pharmacological and non-pharmacological therapies. In this regard, cardiac MRS is expected to grow in importance, as it might estimate, in a non-invasive fashion, the energetic response to metabolic therapies and provide useful surrogate prognostic markers. Finally, future studies shall address Cr supplementation treatment only to HF patients with assessed Cr deficiency, by measuring the absorbed Cr content compared to the treatment dose.

## Central illustration

Failing heart shows a decreased creatine (Cr) content and creatine kinase (CK) activity, as well as a reduced expression of Cr transporter. Phosphocreatine/adenosine triphosphate ratio (PCr/ATP), an index of efficiency of cardiac energy metabolism, is also lower than normal value (< 1.60). Differently from Cr supplementation, treatment with PCr has showed to reduce short-term mortality, to improve left ventricular ejection fraction and to decrease the risk of major arrhythmias [61]. However, further investigation is needed for long-term treatment.

## Supplementary information

Below is the link to the electronic supplementary material.

Supplementary file1 (DOC 18 KB)

## Data Availability

Not applicable.

## Abbreviations

ADP: Adenosine diphosphate
ATP: Adenosine triphosphate
AGAT: L-arginine:glycine amidinotransferase
Cr: Creatine
CK: Creatine kinase
CrT: Creatine transporter
GA: Guanidinoacetate
GAMT: S-adenosyl-L-methionine:guanidinoacetate N-methyltransferase
HF: Heart failure
MRS: Magnetic resonance spectroscopy
PCr: Phosphocreatine
