# Supplementary material for: Creatine deficiency and heart failure
Source: Heart Fail Rev. 2021 Oct 7;27(5):1605–16. doi: 10.1007/s10741-021-10173-y (PMC9388465; doi:10.1007/s10741-021-10173-y)
Supplement: Supplementary file 1 — Supplementary file1 (DOC 18 KB) [file 10741_2021_10173_MOESM1_ESM.doc]

**References for Table 1**

1. Andreev NA, Andreeva TN, Bichkov IV. Effect of creatine phosphate in congestive heart failure. Curr Ther Res. 1992;51:649-660.
2. Andrews R, Greenhaff P, Curtis S, Perry A, Cowley AJ. The effect of dietary creatine supplementation on skeletal muscle metabolism in congestive heart failure. Eur Heart J. 1998;19:617-22. https://doi.org/10.1053/euhj.1997.0767.
3. Cafiero M, Strumia E, Pirone S, Pacileo S, Santoro R. [The efficacy of creatine phosphate in the treatment of patients with heart failure. Its echographic evaluation after acute and protracted treatment]. Clin Ter. 1994;144:321-8. Italian. PMID: 8205755.
4. Carvalho AP, Rassi S, Fontana KE, Correa Kde S, Feitosa RH. Influence of creatine supplementation on the functional capacity of patients with heart failure. Arq Bras Cardiol. 2012;99:623-9. https://doi.org/10.1590/s0066-782x2012005000056.
5. Cornelissen VA, Defoor JG, Stevens A, Schepers D, Hespel P, Decramer M, Mortelmans L, Dobbels F, Vanhaecke J, Fagard RH, Vanhees L. Effect of creatine supplementation as a potential adjuvant therapy to exercise training in cardiac patients: a randomized controlled trial. Clin Rehabil. 2010;24:988-99. https://doi.org/10.1177/0269215510367995.
6. Du XH, Liang FY, Zhao XW. [Effects of phosphocreatine on plasma brain natriuretic peptide level in elderly patients with chronic congestive heart failure]. Nan Fang Yi Ke Da Xue Bao 2009;29:154–5. PMID: 19218138.
7. Ferraro S, Codella C, Palumbo F, Desiderio A, Trimigliozzi P, Maddalena G, Chiariello M. . Hemodynamic effects of creatine phosphate in patients with congestive heart failure: a double blind comparison trial versus placebo. Clin Cardiol. 1996;19:699-703. https://doi.org/10.1002/clc.4960190905.
8. Fumagalli S, Fattirolli F, Guarducci L, Cellai T, Baldasseroni S, Tarantini F, Di Bari M, Masotti G, Marchionni N. Coenzyme Q10 terclatrate and creatine in chronic heart failure: a randomized, placebo-controlled, double-blind study. Clin Cardiol. 2011;34:211-7. https://doi.org/10.1002/clc.20846.
9. Gordon A, Hultman E, Kaijser L, Kristjansson S, Rolf CJ, Nyquist O, Sylvén C. Creatine supplementation in chronic heart failure increases skeletal muscle creatine phosphate and muscle performance. Cardiovasc Res. 1995;30:413-8. PMID: 7585833.
10. Grazioli I, Strumia E. Terapia con creatina fosfato nel paziente con insufficienza cardiaca in fase di scompenso: studio policentrico. G Ital Ric Clin Ter. 1989;10:39-45.
11. Grazioli I, Melzi G, Strumia E. Multicentre controlled study of creatine phosphate in the treatment of heart failure. Curr Ther Res. 1992;52:271-280.
12. Kuethe F, Krack A, Richartz BM, Figulla HR. Creatine supplementation improves muscle strength in patients with congestive heart failure. Pharmazie. 2006;61:218-22. PMID: 16599263.
13. Wang FR, Zheng X. Effects of phosphocreatine on plasma brain natriuretic peptide level and left ventricular function in patients with heart failure. Liaoning: Affliated Hospital, Chinese Medicine University. PJCCPVD 2008;16:29-31.
14. Ying W, Chen Y. Efficiency of Creatine Phosphate Sodium and Hyzaar in hypertensive patients with diastolic dysfunction. Heart 2013;99:A222.
